# Consumption, relative deprivation and mental health: evidence from hedonic consumption

**DOI:** 10.3389/fpubh.2025.1511547

**Published:** 2025-02-10

**Authors:** Hao Li, Weihong Zeng

**Affiliations:** Jinhe Center for Economic Research, Xi'an Jiaotong University, Xi'an, Shaanxi, China

**Keywords:** relative deprivation, hedonic consumption expenditure, consumption inequality, reduced consumption, public mental health, China

## Abstract

**Introduction:**

In recent years, relative deprivation related to consumption has sparked intense debate, particularly as the COVID-19 pandemic caused incalculable economic losses worldwide. However, the relationship between relative deprivation related to consumption and mental health remains largely unexplored. This study investigates how both vertical (household-level) and horizontal (household-to-household) relative deprivation related to consumption affect mental health, with a focus on hedonic consumption, and identifies underlying channels and moderating factors.

**Methods:**

We analyze data from the China Family Panel Studies (CFPS) covering 88,144 observations from 2010 to 2018. Hedonic consumption is measured through expenditure on items such as jewelry, antiques, and entertainment, while consumption inequality is assessed using the Kakwani index. Mental health is evaluated using the CES-D and Kessler 6 scales. Ordinary least squares (OLS) and two-stage least squares (2SLS) methods are employed.

**Results:**

Our findings show that reductions in hedonic consumption negatively impact mental health, with involuntary and sudden declines resulting in more pronounced deterioration. Furthermore, greater consumption inequality exacerbates mental health issues, and perceived unfair treatment amplifies this effect. Additionally, self-perception and trust levels are identified as key channels through which these effects. Furthermore, cultural variations and social capital are moderating roles to diminish the adverse mental health.

**Conclusion:**

This study advances our understanding of how relative deprivation related to consumption affects mental health and offers valuable insights for policymakers and practitioners aiming to address these challenges.

## 1 Introduction

Adverse psychological states often arise from external stressors, with certain negative factors in the social environment contributing to sensations such as relative deprivation. Central to the concept of relative deprivation is comparison, which involves vertical comparisons between one's current situation and past circumstances, as well as horizontal comparisons between an individual and a reference group (1, 2). When individuals perceive themselves as disadvantaged or falling short of their desired outcomes, they are likely to experience emotions such as loss, frustration, anger, and even resentment, which constitute a sense of relative deprivation (3). Prolonged exposure to negative emotional stimuli exacerbates feelings of victimization, and the accumulation of such emotions over time increases the risk of psychological problems (4). In today's consumer-driven society, people are not only addicted to consumption, but also easier to learn about other people's consumption through various channels, such as social media. Therefore, both vertical and horizontal comparisons related to consumption are intensified, further worsening psychological distress when individuals experience relative deprivation. Despite this, few empirical studies have explored the health effects of relative deprivation related to consumption.

A comprehensive study on relative deprivation related to consumption is essential for several reasons. Firstly, in micro-economics, income can only bring utility to a certain consumer if it is spent. Therefore, consumption is more directly related to people's mental health. Besides, consumption can also be considered as a function of permanent (visible) or transitory (hidden) income, wealth and other social resources. Thus, estimating relative deprivation related to consumption is a more comprehensive way to measure social welfare (5). Secondly, consumptions are considered to be a more comprehensive measure of individuals' economic resources and are superior to income for explaining mental health (6, 7), especially in the long-term (8). Therefore, relative deprivation related to consumption is crucial for understanding variations in mental health. Thirdly, in reality, reported consumption data consists of hundreds of detail sub-items while total income typically only include wage, transfer, property and operational income. Hence, income is much easier to be manipulated and always being controversial. But consumption has more structural and is statistically more reliable (5).

Mental health is a major component of overall health. The World Health Organization's (WHO) concept of “No health without mental health” has become widely accepted among mental health professionals (9). The prevalence of mental disorders in China is high, resulting in serious economic and social burdens. It is estimated that there are about 130 million people aged 18 years or older living in China who suffer from a mental disorder (10). However, most people with mental health problems do not have access to treatment (11).

Several factors impact mental health, including stress, social support, and socioeconomic status (12, 13). Among these, stress plays a key role and interacts with other factors (12). Stress is an emotional state frequently experienced by individuals; it generally refers to a state of psychological tension that individuals experience in work, life, interpersonal relationships, and personal responsibilities. It can occur when individuals perceive their environmental demands to be taxing or beyond their available coping resources, thereby endangering their overall wellbeing (14). Besides, stress is considered as a universal key factor related to mental health throughout developing countries like China (15). One critical but often overlooked stressor is relative deprivation related to consumption. On the one hand, consumption inequality reflects horizontal comparisons between peers, where individuals may feel deprived and stressed if their peers have greater access to consumer goods (16). Much of the existing research has focused on income inequality, showing that it increases social deprivation, undermines social cohesion, and restricts equal access to public resources, all of which contribute to negative health outcomes (17, 18). On the other hand, reduced consumption spending represents vertical comparisons, where individuals may experience diminished happiness when their current spending falls short of their past spending levels. While many studies have examined income-related economic hardships and their clear association with poor mental health outcomes, such as psychological distress and depression (19, 20), less attention has been paid to other forms of economic hardship, like reduced consumption expenditure.

Given the gaps in existing research, this paper aims to explore the relationship between relative deprivation related to consumption, both vertical and horizontal, and mental health using nationally representative longitudinal data. Specifically, the study utilizes data from the China Family Panel Studies (CFPS) from 2010 to 2018 to assess the impact of reduced consumption expenditure (vertical comparisons) and consumption inequality (horizontal comparisons) among hedonic consumption categories on mental health as hedonic consumption has more direct impact on mental health and often serves as an extra consumption compared to other consumption items.

This study makes several contributions to the literature on consumption and mental health, with a focus on hedonic consumption. While much of the existing research has concentrated on the effects of increased hedonic consumption on mental health, the consequences of reduced hedonic consumption remain underexplored. Moreover, the relationship between consumption inequality and mental health is also understudied, despite its importance. In sum, this study provides empirical evidence to enhance the understanding of how relative deprivation related to consumption influence mental health, particularly within the Chinese context.

The remainder of this study is organized as follows. We first review related literature and propose the hypothesis in Section 2. In Section 3, we introduce materials and methods. In Section 4, we report our results, and we discuss and conclude in Section 5.

## 2 Related literature

Scholars have claimed that the consumption of certain products can lead to relevant feeling (21). For example, the purchase of hedonic products may make consumers feel enjoyable (22). Experiential consumption, such as traveling, watching movies, and eating outside, is associated with improved mental health (23, 24).

Consumption expenditures can be categorized into three types based on their purpose: basic consumption, developmental consumption, and hedonic consumption (25). Basic consumption, namely, mainly meet individuals' basic needs, such as food and clothes expenditures. On this level, individuals need to purchase these kinds of “must-have” products to fulfill the physical requirement for human survival (26). Developmental consumption includes spending on medical insurance and education, aimed at enhancing human capital. Hedonic consumption refers to expenditures on products that provide pleasure, fun, and enjoyment, appealing to the senses (27), such as purchasing jewelry, antiques, musical equipment, and cultural and entertainment expenditures, and they are linked to positive feelings (28). According to Maslow's hierarchy of needs, human behavior is motivated by the pursuit of increasingly higher needs once basic needs are met (29). Therefore, individuals are more likely to prioritize necessities over hedonic consumption when they faced deprivation.

Hedonic consumption can be considered extra consumption, which is possible after the basic and developmental needs are fulfilled. Most studies support the positive effect between increased hedonic consumption spending and mental health (22). Thus, the possession and consumption of more hedonic products represents the surest perceived route to personal happiness and well-being. However, there is limited literature on the effects of reduced consumption on mental health, particularly concerning hedonic consumption, despite the importance of this issue. One study points that consumers feel stressed if their consumption of certain products becomes repressed, and the stress could be alleviated by consuming corresponding products (30). Specially, when consumers face a threat, whether actual or potential, it disrupts their routines, norms, beliefs, and behaviors. Therefore, we propose the following hypothesis:

**Hypothesis 1a:** Reductions in hedonic consumption expenditure worsens people's mental health.

It has long been suggested that high inequality may negatively affect individuals' health, as feelings of relative deprivation can increase stress and frustration (31). Inequality increases social uncertainty and the perception of threat in social interactions, generating negative psychological consequences (32). Consumption inequality is a public bad, and from a welfare perspective, it may be more worrying than income inequality (5). First, as consumption is a function of income, income inequality, to some extent, results in consumption inequality (33), which a plethora of studies exist that focus on income inequality and mental health (34). Second, consumption inequality is a form of relative deprivation. It reflects the imbalanced distribution of consumption resources, capabilities, and opportunities, influenced by individual differences in abilities as well as disparities in resources and opportunities (35). A sense of relative deprivation can trigger individuals to adopt negative coping styles (36), thus influence mental health. Third, empirical evidence based on data from the Urban Household Survey suggests that consumption inequality in urban China has increased by 67%, surpassing the original figure of 36% reported by raw data (5). Consumption inequality exhibits a greater magnitude in China, whereas income inequality is less pronounced, making it easier to detect significant results in related studies. Therefore, we propose the following hypothesis:

**Hypothesis 1b:** Consumption inequality among hedonic items natively affect people's mental health.

Different types of reduced hedonic consumption have different effects on mental health. The study shows that individual vulnerability factor that may increase the propensity for depression in individuals who are treated is heightened reactivity to unpredictable threat (U-threat). U-threat is a specific type of stressor that is ambiguous in its timing, intensity, frequency, while predictable threat (P-threat) is a type of imminent and immediately present stressor signaled by a brief, discrete cue. Moreover, U-threat is more sustained and aversive as it diminishes one's ability to predict and effectively prepare for future events, and thus elicits a generalized feeling of hypervigilance and apprehension (37). Therefore, we propose the following hypothesis:

**Hypothesis 2a:** Unpredictable declines in hedonic consumption expenditure could result in greater mental disorders.

Additionally, the study shows that exposure to just one life shock will result in a greater risk of mental disorders (38). Conversely, chronic stress decreases the susceptibility of individuals against acute stressful events. People are “steeled” or “hardened” by ongoing chronic stress, reducing the impact of later acute negative events (39). For example, chronic economic hardship may reduce susceptibility to acute stressful events such as a temporary pay cut. Therefore, we propose the following hypothesis:

**Hypothesis 2b:** Individuals experience greater distress when faced with sudden, one-time hedonic consumption expenditure decline rather than chronic decline.

Duesenberry points that consumers are reluctant to voluntarily reduce their prior consumption expenditure, a phenomenon known as the ratchet effect (40). In other words, individuals experience poorer mental health when their life situation deteriorates compared to when it improves. Additionally, Oral and Thurner point that by rejecting excessive consumption, some individuals have started to moderate their consumption levels in order to enhance mental well-being (41). However, this satiation is not likely to happen in developing countries. Therefore, we propose the following hypothesis:

**Hypothesis 2c:** Involuntary hedonic consumption expenditure decline could result in greater mental disorders.

Consumption inequality is a form of relative deprivation. Perceived unfairness is an essential ingredient that can not only breed relative deprivation but also modify the extent to which it is related to adverse outcomes of well-being. The sense of unfairness is cognitive evaluation, which refers to the perception of an individual on disadvantageous situation derived from social comparison. People believe that their disadvantageous situation is unfair and they deserve better treatment. This experience can generate a sense of shame, loss and inferiority, which further undermines psychological stability and can eventually lead to lower levels of mental health (42). Therefore, we propose the following hypothesis:

**Hypothesis 2d:** The experience of unfair treatment amplifies the impact of consumption inequality on mental health.

Research on the underlying channels is limited. Reduced consumption expenditure often reflects economic deprivation, particularly in developing countries like China, where it can lead to declines in trust, especially trust in strangers. Trust is especially relevant in situations involving conflicts of interest (43). Factors such as social injustice, conflicts with government officials, and deprivation significantly reduce trust (44, 45), which in turn can lead to mental health problems. Regarding consumption inequality, one line of argument centers on the mediating role of self-perception (46). Self-perception refers to the judgment of the integrity of self-knowledge formed by people *via* the judgment of their own ability and value and the degree of respect for an individual's sense of self that is triggered by this judgment (47). The external context and internal emotions influence the direction of an individual's self-esteem perception change. They can be divided into two categories: self-esteem perception enhancement and self-esteem perception threat. Self-esteem perception enhancement occurs when individuals receive positive feedback and have a positive evaluation of their current competence and value. On the other hand, when individuals receive negative information that triggers negative emotions, they may experience a state of denying their competence and value, which is known as a self-esteem perception threat. Briefly, individuals who feel a sense of material inequality may come to doubt their value and significance as individuals. They may believe - whether accurately or not - that persons around them regard them as unworthy. These negative reflected appraisals and unfavorable social comparisons may undermine mental health (48). Therefore, we propose the following hypothesis on potential channels:

**Hypothesis 3a:** Decreased trust levels may play a mediating role in the relationship between reduced consumption expenditure and mental health.

**Hypothesis 3b:** Lowered self-perception may play a mediating role in the relationship between consumption inequality and mental health.

Cultural variations and social capital are well known for alleviating adverse mental health effects. On one hand, religion, as an important aspect of cultural variation, has been found to positively influence mental health (49, 50). Religious beliefs can improve mental health by fostering morality, enhancing coping mechanisms, encouraging respect for diversity, and promoting social connectedness. For instance, through shared beliefs and practices, congregants often achieve a high level of social integration, which benefits mental health (51). Additionally, religious beliefs influence how individuals handle stress, suffering, and life challenges (52), enhancing acceptance and resilience in the face of adversity (53). On the other hand, individuals are embedded in society, and social capital significantly impacts various aspects of life, including mental health. Social capital refers to the relationships and interactions between individuals and groups. It can be understood and measured at both individual and collective levels (54). At the individual level, social capital is a personal resource derived from social networks, providing better access to information, services, and support (55). At the collective level, social capital arises within communities and neighborhoods, where it is viewed as a collective property. Research indicates that social capital is associated with better general health and well-being (56). Therefore, we propose the following hypothesis on potential moderators:

**Hypothesis 4a:** Religious beliefs could diminish the negative effect of relative deprivation related to consumption on mental health.

**Hypothesis 4b:** Social capital could diminish the negative effect of relative deprivation related to consumption on mental health.

**Figure 1:**
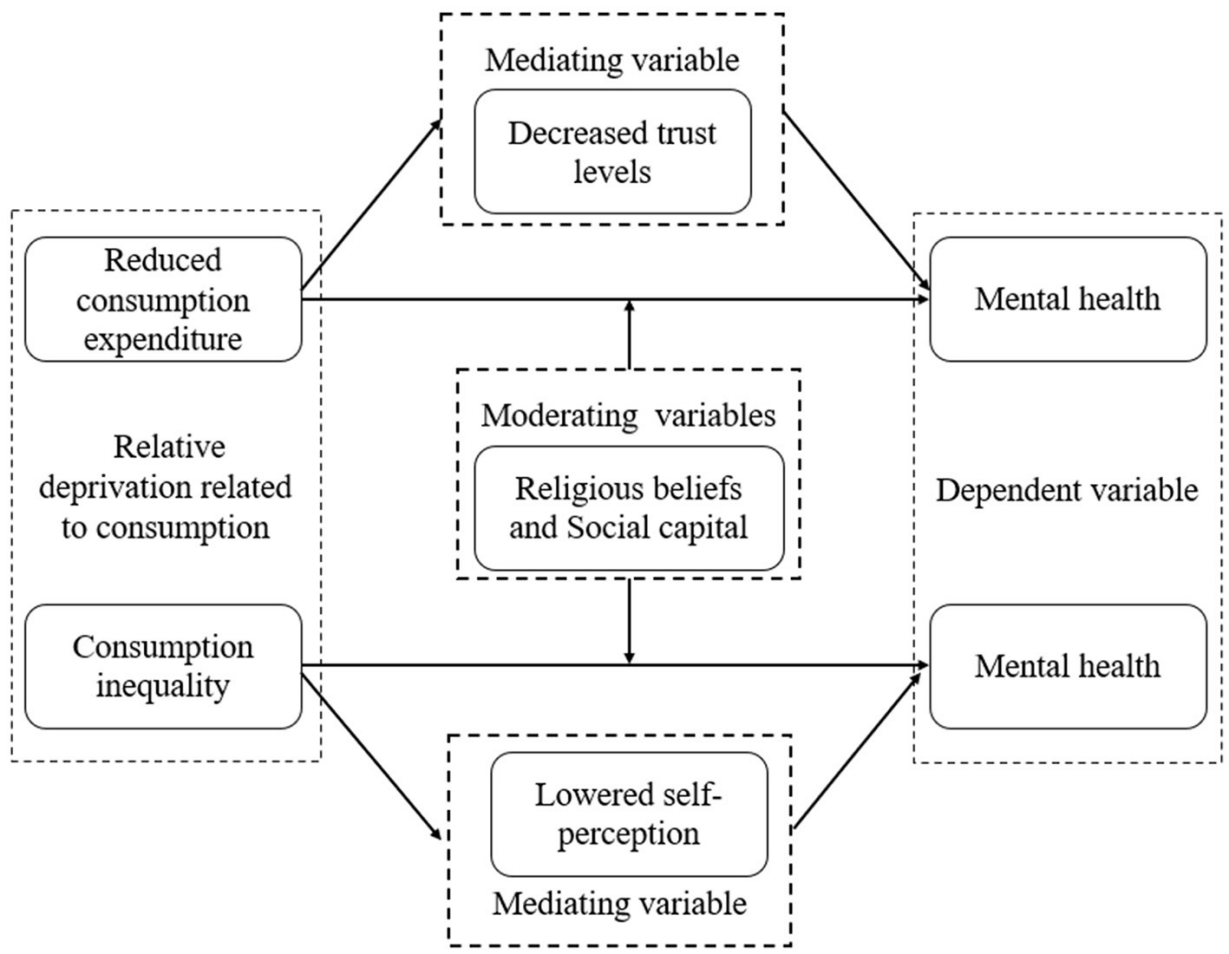
Theoretical framework.

In sum, the theoretical framework of this study is depicted in Figure 1.

## 3 Materials and methods

### 3.1 Data

The China Family Panel Studies (CFPS) is a nationally representative longitudinal survey of Chinese communities, families, and individuals launched in 2010 by Peking University. The CFPS surveyed respondents in sampling units in 25 provinces, a sampling frame which represents 95% of the Chinese population. To generate a nationally and provincially representative sample, the CFPS adopted a “Probability-Proportional-to-Size” (PPS) sampling strategy with multi-stage stratification and carried out a three-stage sampling process. The first stage was the Primary Sampling Unit, in which county level units were randomly selected. In the second stage, village level units (villages in rural areas and neighborhoods in urban areas) were selected. In the third stage, households from the village level units were selected according to the systematic sampling protocol of the study.

The data was obtained from five waves (2010, 2012, 2014, 2016, and 2018) of CFPS. The analysis was conducted among people who left the school. Here, we would like to illustrate that, our dependent variable comes from the individual dataset, and independent variable comes from the family dataset. Unlike the concept of “family” in western society, the Chinese family has a strong undertaking function. Western families have strict boundaries, couples are the main axis of them, and the birth and raising of children is the main content of family life. However, a Chinese family has long-term continuity and serves as a unit to organize other activities. In Chinese society, individual investment, education, and employment decisions not only concern individuals but also affect the development and career prospects of the entire family. The relationships that individuals establish in society also extend to other members of the family that the individuals belong to. Chinese people often engage in economic activities and social interactions on a household basis (57). Thus, it is very difficult and impractical to separate individuals from their families in the context of our study.

### 3.2 Measures

#### 3.2.1 Mental health

Mental health comes from the individual dataset. We unify the metrics and standardized the mental health scores with a mean of zero and a standard deviation of one because CFPS collected two mental health scales in different years.

CFPS collected the Kessler 6 rating scale (K6) in wave 2010 and wave 2014. The K6 instrument, developed by Kessler et al., includes six questions about negative emotions and mental status experienced during the past period (58).

The K6 instrument consists of the following six questions to measure the symptoms of depression and mental disorders:

(1) During the past week, how often did you feel depressed that nothing could cheer you up?

(2) During the past week, how often did you feel nervous?

(3) During the past week, how often did you feel restlessness that nothing could calm you down?

(4) During the past week, how often did you feel futureless?

(5) During the past week, how often did you feel difficult that everything was an effort?

(6) During the past week, how often did you feel meaningless?

Individuals have five response options, each corresponding to a score ranging from one to five: none of the time (one point), sometimes (two points), half the time (three points), often (four points), and almost every day (five points). Therefore, higher scores are associated with more frequent depressive symptoms and poorer mental health status in the past week.

The Center for Epidemiologic Studies Depression scale (CESD) was initially developed by Laurie Radloff for research use (59). CFPS collected both CESD 20 in wave 2012 and CESD 8 in wave 2016 wave 2018. The CESD 20, a longer length of time, is necessary for respondents to complete the survey and, thus, may reduce the respondents' motivation to participate (60)^1^. Shortened versions of the CESD have been developed (61), and the most commonly used abbreviated version is CESD 8 (62).

The CESD measures include a set of questions to measure the symptoms of depression and mental disorders, such as: the respondents were asked about how often they had the following feelings over the past week:

(1) How often did you feel depressed?

(2) How often did you feel difficult that everything was an effort?

(3) How often did you feel restless?

(4) Did you enjoy life?

(5) Did you feel you could not get going?

(6) How often did you feel lonely?

(7) How often did you feel happy?

(8) How often did you feel sad?

For each question, individuals could choose one of the following answers: never (one point), sometimes (two points), often (three points), or most of the time (four points). Among them, we performed reverse scoring on question 4 and 7 to ensure consistent evaluation criteria. Therefore, higher scores are associated with more frequent depressive symptoms and poorer mental health status in the past week.

The advantage of using CFPS self-reported mental health is that the recall period is only for one week, which is much closer to the interview date than most other survey data. Therefore, the respondents are less likely to have biased recall on their mental health problems when recalling the feelings.

#### 3.2.2 Reduced hedonic consumption expenditure

The independent variable in this study is household consumption. We employ expenditures on jewelry, antiques, musical equipment, and cultural and entertainment activities to measure hedonic consumption expenditure. Additionally, for robustness checks, we use expenditures on traveling, watching movies, and eating outside to measure experiential consumption expenditure because experiential consumption is perceived to be more unique (63), more able to assist individuals develop their ideal-self (64), and offers greater happiness to individuals (65, 66). All consumption expenditures are derived from family dataset. The independent variable of reduced hedonic consumption expenditure is established as following two steps:

Step 1: calculating the ratio

We first calculate the ratio of hedonic consumption expenditure relative to total consumption expenditure (see Equation 1). Notably, we use relative numbers instead of absolute numbers as absolute figures may not fully capture changes in hedonic consumption expenditure. For example, if a household spent 10,000 yuan on hedonic consumption in 2012 and 12,000 yuan in 2014, a superficial analysis might suggest an increase in hedonic consumption. However, considering changes in family income, relative consumption offers a more accurate representation. The use of relative numbers holds the implicit assumption that individuals, or households for that matter, do not try to manipulate their relative position in society and passively accept the rank that is awarded to them on the basis of how their total level of income/consumption compares to that of others (67).


(1) $$\begin{array}{l} Ratio_{ft}=\frac{Hedonic\;Consumption\;Expenditure_{ft}}{Total\;Consumption\;Expenditure_{ft}} \end{array}$$

Step 2: constructing the independent variable

Building on Step 1, we construct the independent variable by subtracting the proportion of hedonic consumption expenditure in the previous year from that in the subsequent year (see Equation 2). This yields a dummy variable named “Reduction” with a value of 1 indicating decrease hedonic consumption, as depicted in Table 1.


(2) $$Reduction_{ft}=\{\begin{array}{c} 1\;if\;Ratio_{ft+1}-Ratio_{ft}<0\\ 0\;if\;Ratio_{ft+1}-Ratio_{ft}\geq 0 \end{array}$$

**Table 1:** Variable definition and descriptive statistics.

**Variables**	**Definition**	**Mean (SD)**	**Min**	**Max**
Mental health	Standardized mental health	0(1)	−1.06	5.56
Reduction_ft	1: Reduced hedonic consumption expenditure; 0: Otherwise	0.54(0.50)	0	1
*IneqC*_*ft*_	Calculated using Kakwani Index	0.65(0.40)	0	1
Age	Individual's age	49(15)	20	102
Gender	1: Male; 0: Female	0.50(0.50)	0	1
Educ status	1: Illiterate; 2: Primary school; 3: Junior high school; 4: Senior high school; 5: Junior college; 6: University degree; 7: Master degree; 8 PhD degree	2.45(1.28)	1	8
Marital status	1: Married; 0: Otherwise	0.84(0.36)	0	1
Hukou status	1: Urban hukou; 0: Otherwise	0.25(0.43)	0	1
Employment	1: Employment; 0: Otherwise	0.67(0.47)	0	1
Cohort	1: Born in the Great Famine; 0: Otherwise	0.04(0.20)	0	1
Attitude gap	0: Income inequality is not severe; 10: Income inequality is severe	6.84(2.52)	0	10
Smoking status	1: Yes; 0: No	0.30(0.46)	0	1
Drinking status	1: Yes; 0: No	0.16(0.37)	0	1
Social interaction attitude	1: Not important; 5: Important	3.02(0.59)	1	5
Chronic diseases	1: Yes; 0: No	0.12(0.33)	0	1
Self-reported health	1: Very good; 2: Good; 3: Somewhat healthy; 4: Normal; 5: Very bad	3.15(1.24)	1	5
Inpatient	1: Inpatient; 0: Otherwise	0.12(0.32)	0	1
Income	Annual net individual total income (RMB yuan)	8,106(15,930)	1	100,000
Savings	Annual net household total savings (RMB yuan)	30,450(67,126)	1	500,000
Debt status	1: Yes; 0: No	0.21(0.41)	0	1
Family size	Number of family members	4(2)	1	24
GDP	Provincial GDP (RMB hundred million yuan)	29,557(21,243)	2,131	99,945

#### 3.2.3 Consumption inequality

Measurement indexes of consumption inequality include Gini, Theil, and Kakwani indexes. Considering the study's focus and the Kakwani index's normality and quantitative rigidity advantages, this study used the Kakwani index to assess consumption inequality (68). In this context, respondents' families form a group with *n* samples, and their corresponding consumption vector is denoted as *X* = (*x*_1_, *x*_2_, , *x*_*n*_). The consumption elements *x*_*f*_ are sorted in ascending order, namely *x*_1_ ≤ *x*_2_ ≤ ≤ *x*_*n*_. $u_{x_{f}}^{+}$ is the average consumption of the samples that consumption exceeds *x*_*f*_ in the vector *X*. $r_{x_{f}}^{+}$ is the ratio of the samples that consumption exceeds *x*_*f*_ in the vector *X* in the whole sample, and *u*_*X*_ is the average consumption of all samples. Lastly, the Kakwani index is calculated using the following formula (see Equation 3):


(3) $$\begin{array}{l} IneqC_{ft}=\frac{1}{nu_{X}}\overset{n}{\underset{k=f+1}{\sum }}\left(x_{k}-x_{f}\right)=r_{x_{f}}^{+}\left[\frac{\left(u_{x_{f}}^{+}-x_{k}\right)}{u_{X}}\right] \end{array}$$

In Equation 3, *IneqC*_*ft*_represents the degree of consumption inequality for a household compared to other households within the same community. Higher and lower values indicate greater and weaker consumption inequality, respectively, in the respondent's household. To ensure the robustness of our results, we also use the Gini coefficient and Theil index as alternative measures of consumption inequality in the robustness analysis (69).

#### 3.2.4 Covariates

We include controls for individual demographic, household, and provincial characteristics known to affect both consumption and mental health. First, we include individual and household factors such as age, age-squared, gender, *Hukou* status, education level, employment status, marital status, chronic diseases, self-reported health, and family size. Additionally, social interaction attitudes, such as the strength and frequency of social interactions, are also linked to mental health (70). We also control for risky health behaviors, such as smoking and drinking, as the evidence has demonstrated that risky health behaviors are associated with mental health (71). Wealth is another crucial factor influencing both consumption and mental health. Thus, we account for the natural logarithm of annual net individual income, the natural logarithm of annual net household savings, and debt status. At the provincial level, we control for the natural logarithm of GDP. Adverse childhood experiences have been associated with various long-term negative outcomes, including consumption patterns and mental health (72, 73). Therefore, we control for individuals born between 1959 and 1961 to account for the impact of the Great Famine in China. Additionally, we include controls for inpatient status to capture short-term shocks and attitudes toward inequality in China to reflect long-term attitudes affecting both mental health and consumption behaviors.

Table 1 presents the characteristics of all covariates. As with previous work (5), the mean value of consumption inequality is 0.65, indicating consumption inequality is a relatively severe issue in China. The mean value “Reduction” is 0.54, implying that half of the people reduced their hedonic consumption expenditure. The average age of participants is 49, with 50% being female. Rural residents make up 75% of the sample. Marriage and employment rates are high, with 84% married and 67% employed. The proportion of inpatients and those born between 1959 and 1961 is low. The mean value of educational attainment is 2.45, indicating that the average level of education is junior high school graduation. Risky health behaviors are also notable, with 30% of participants reporting smoking and 16% reporting drinking. The average self-reported health score is 3.15, and 12% of participants have a chronic condition. Furthermore, the average “Attitude gap” is 6.84, ranging from 0 to 10, showing that most individuals believe inequality is a severe problem. The average “Social interaction attitude” is 3.02, showing that most individuals believe social interaction is important. At the household level, the average individual income is 8,106 yuan, while the average household savings amount to 30,450 yuan. Additionally, 21% of individuals report having debt, and the average family size is four. At the provincial level, the average GDP is 29,557 hundred million yuan.

### 3.3 Statistics analysis and model specification

#### 3.3.1 Baseline model

We estimate the following regression model for consumption and mental health (see Equation 4):


(4) $$y_{it}=\beta_{0}+\beta_{1}Reduction_{ft}/IneqC_{ft}+\beta_{2}X_{*t}+\pi_{t}+{\text{λ}}_{f}+{\text{λ}}_{p}+\varepsilon_{it}$$

where *y*_*it*_ represents individual *i*'s mental health in year *t*. *Reduction*_*ft*_ represents whether hedonic consumption expenditure reduced in household *f* at year *t*. *IneqC*_*ft*_ represents consumption inequality among hedonic items in household *f* at year *t*. The vector $X_{*t}$ consist of control variables at individual-level, family-level and provincial-level, respectively. π_*t*_, λ_*f*_, and λ_*p*_ represent the fixed effects of the survey year, household-level, and provincial-level to control for unobservable factors. ε_*it*_ is the error term. All standard errors are the robust standard errors. β_1_ is the main coefficient of interest, β_1_>0 indicates that people who are exposed to reduced hedonic consumption may have worse mental health, and people who are exposed to higher consumption inequality among hedonic items may have worse mental health.

#### 3.3.2 Endogeneity and instrumental variable

Estimating the impact of reduced hedonic consumption on mental health creates a potential for endogeneity.

Consumption expenditures are likely to be endogenous for various reasons. One potential source of endogeneity, in the context of our study, is omitted variable bias. There are several unobservable factors that are likely to be correlated with both consumption expenditure and mental health and, thus, in a multivariate regression framework, it is difficult to rule out more than one omitted variable, making it impossible to clearly predict the direction of bias. One source of omitted variable bias, for example, is personality traits. Personality traits influence the consumption expenditure (74). At the same time, the psychological well-being and mental health of a person accepts an impact of personality traits (75). The exclusion of this variable in a model examining the relationship between consumption expenditure and mental health could potentially result in either underestimation (downward bias) or overestimation (upward bias), depending on individual's personality traits.

While the fixed effect model is generally known to be effective in addressing omitted variable bias, endogeneity may also emerge from potential measurement error and simultaneity bias. Measurement error can result from memory bias. This could also result in the fixed effect estimates being upward or downward biased, depending on whether people report above or below the real consumption expenditures. Indeed, simultaneity bias is the main concern of our study (76). For example, stress could increase consumption expenditures, especially increase in hedonic consumption expenditures (77).

For consumption inequality, although the Kakwani index possesses properties such as being dimensionless, normally distributed, and transfer invariant (78), minimizing concerns about potential measurement error, omitted variables—such as personality traits and risk preferences—are likely to be correlated with both consumption inequality and mental health.

Case and Katz, as well as Duflo and Saez, suggest using the average exogenous characteristics of group-level variables as instruments for endogenous independent variables (79, 80). In the context of this study, this refers to the average exogenous characteristics from higher administrative levels. Drawing on the research Yin et al. (81), we use the reduced hedonic consumption expenditure of other households within the same community and the consumption inequality among hedonic items in other households within the same county as instrumental variables.

As for the former instrumental variable, a village-rooted and kinship-based organization has been the central mechanism to sustaining China's society (82). Communities, being the smallest social unit of rural society, function as acquaintance-based society, characterized by information symmetry and social network access (83), where people generally know each other well. In urban areas, communities are naturally grouped by housing prices after housing reforms, with people of similar consumption levels typically living in the same communities. On the other hand, previous literature has widely used community-level measurements, which exclude individual and family characteristics, as instrumental variables (81). Since hedonic consumption is typically a private matter, the reduced hedonic consumption expenditure of other households within the same community does not directly affect an individual's mental health, making community-level exogenous variation a valid instrumental variable.

Regarding the second instrumental variable, counties are the basic spatial units of county-level administrative regions in China. As the link between urban and rural areas, counties play a crucial role in transitioning from the dual economic structure to promoting China's broader economic and social development. County-level economies exhibit convergence, implying that consumption inequality within the same county also converges. Moreover, the spatial distribution among communities within a county suggests that consumption inequality in other communities is unlikely to directly affect an individual's mental health. Thus, county-level exogenous variation also serves as a feasible instrumental variable.

Moreover, we adopt higher moment instruments approach proposed by Lewbel to make an internal instrumental variable as the method without relying on external factors (84). Lewbel (85) suggests using the cubic relationship between independent variables and the mean value of their higher moments. This approach, originally designed for measurement error models, has proven useful in dealing with general correlated-regressor errors and multilevel models. Following this framework, we use two Lewbel's IVs (see Equations 5, 6).


(5) $$Lewbel\;IV_{Reduction_{ft}}={\left(Reduction_{ft}-\bar{Reduction_{community-level\;t}}\right)}^{3}$$

(6) $$\begin{array}{l} Lewbel\;IV_{IneqC_{ft}}={\left(IneqC_{ft}-\bar{IneqC_{county-level\;t}}\right)}^{3} \end{array}$$

where $\bar{Reduction_{community-level\;t}}$ is the mean value of reduced hedonic consumption at the community level, and $\bar{IneqC_{county-level\;t}}$ is the mean value of consumption inequality among hedonic items at county level.

#### 3.3.3 Channels analysis

We employ the questions that “*Should most people be trusted?”* to measure trust levels, and use the question that “*Last year, compared to the norm, was your family's social status/total income higher or lower?”* to measure self-perceptions. The former one is a dummy variable with 0 represents lower trust level, and the later one is a discrete variable with higher scores indicate better self-perceptions.

#### 3.3.4 Moderating analysis

We assess religious beliefs using a single question: “*Do you have religious beliefs?”* This is a binary variable, where 0 indicates no religious beliefs. For social capital, we evaluate it at both individual and collective levels. At the individual level, social capital is assessed using education level and annual net individual income, as these are key personal resources. At the collective level, we draw from the literature (86) and use generalized trust, social participation, and emotional and instrumental social support as moderators. Specifically, we measure these aspects with the following questions: “*Do you feel that people around you are trustworthy?”, “How many visitors did you receive during Chinese New Year?”*, and “*How often do you chat with relatives and neighbors?”* Higher values reflect greater collective social capital. Finally, we aggregate individual-level and collective-level social capital into a combined measure.

## 4 Results

### 4.1 Baseline model

Table 2 presents the baseline results from a vertical comparison within households, exploring the association between reduced hedonic consumption expenditure and mental health among all participants. The OLS regression of Column 1 shows that the coefficient of *Reduction*_*ft*_ is positive and significant at the 1% statistical level, indicating an adverse relationship between reduced hedonic consumption expenditure and mental health. The findings support Hypothesis 1a. In the last two columns, Column 2 presents results based on the traditional instrumental variable (IV) method, while Column 3 reports results using the Lewbel's IV method. Both methods confirm that reduced hedonic consumption expenditure is linked to higher rates of mental disorders in China. Moreover, the *F* statistics are larger than the general standard of 10, indicating that the statistical results reject the original hypothesis of “weak identification of instrumental variable”. Additionally, the *p-*value of underidentification test statistic is 0.00, rejecting the null hypothesis and confirming the validity of the instrument.

**Table 2:** The impact of reduced hedonic consumption expenditure on mental health.

	**OLS**	**IV**	**Lewbel's IV**
*Reduction*_*ft*_	0.009^***^	0.038^***^	0.008^**^
	(0.003)	(0.009)	(0.003)
Age	0.002^***^	0.002^***^	0.002^***^
	(0.001)	(0.001)	(0.001)
Age squared	−0.000^***^	−0.000^***^	−0.000^***^
	(0.000)	(0.000)	(0.000)
Gender	−0.087^***^	−0.087^***^	−0.087^***^
	(0.004)	(0.004)	(0.004)
Educ status	−0.014^***^	−0.014^***^	−0.014^***^
	(0.002)	(0.002)	(0.002)
Marital status	−0.073^***^	−0.073^***^	−0.073^***^
	(0.006)	(0.006)	(0.006)
Hukou status	−0.008	−0.007	−0.008
	(0.008)	(0.008)	(0.008)
Employment	−0.021^***^	−0.021^***^	−0.021^***^
	(0.004)	(0.004)	(0.004)
Cohort	−0.001	−0.001	−0.001
	(0.010)	(0.010)	(0.010)
Attitude gap	0.006^***^	0.006^***^	0.006^***^
	(0.001)	(0.001)	(0.001)
Smoking status	0.004	0.004	0.004
	(0.005)	(0.005)	(0.005)
Drinking status	−0.008	−0.008	−0.008
	(0.005)	(0.005)	(0.005)
Social interaction attitude	0.003	0.003	0.003
	(0.003)	(0.003)	(0.003)
Chronic status	0.059^***^	0.060^***^	0.059^***^
	(0.007)	(0.006)	(0.006)
Self-reported health	0.088^***^	0.088^***^	0.088^***^
	(0.002)	(0.002)	(0.002)
Inpatient	0.053^***^	0.052^***^	0.053^***^
	(0.005)	(0.005)	(0.005)
Log income	−0.002^***^	−0.002^***^	−0.002^***^
	(0.000)	(0.000)	(0.000)
Log savings	0.000	0.000	0.000
	(0.000)	(0.000)	(0.000)
Debt status	0.008	0.008	0.008
	(0.005)	(0.005)	(0.005)
Family size	−0.001	−0.001	−0.001
	(0.002)	(0.002)	(0.002)
Log GDP	−0.111^***^	−0.114^***^	−0.111^***^
	(0.027)	(0.024)	(0.024)
*F*-stat		611	10,835
Underidentification test *P-*value		0.000	0.000
Year FE	Yes	Yes	Yes
Household FE	Yes	Yes	Yes
Provincial FE	Yes	Yes	Yes
Constant	0.896^***^		
	(0.270)		
Observations	88,144	88,144	88,144
*R* ^2^	0.851	0.083	0.084

^**^p < 0.05; ^***^p < 0.01.

Table 3 provides the baseline results from a horizontal comparison between households, examining the association between consumption inequality on hedonic items and mental health. The OLS regression in Column 1 shows that the coefficient of *IneqC*_*ft*_ is positive and significant at the 1% statistical level, indicating that there is an adverse relationship between consumption inequality among hedonic items and mental health, thus supporting Hypothesis 1b. The last two columns present results from the traditional IV method and the Lewbel's IV method, respectively. These results remain robust after addressing endogeneity concerns. Additionally, the *F* statistics and underidentification test are remain robust.

**Table 3:** The impact of consumption inequality among hedonic items on mental health.

	**OLS**	**IV**	**Lewbel's IV**
*IneqC*_*ft*_	0.013^**^	0.028^**^	0.016^**^
	(0.006)	(0.012)	(0.007)
Age	0.002^***^	0.002^***^	0.002^***^
	(0.001)	(0.001)	(0.001)
Age squared	−0.000^***^	−0.000^***^	−0.000^***^
	(0.000)	(0.000)	(0.000)
Gender	−0.087^***^	−0.087^***^	−0.087^***^
	(0.004)	(0.004)	(0.004)
Educ status	−0.014^***^	−0.014^***^	−0.014^***^
	(0.002)	(0.002)	(0.002)
Marital status	−0.073^***^	−0.073^***^	−0.073^***^
	(0.006)	(0.006)	(0.006)
Hukou status	−0.008	−0.008	−0.008
	(0.008)	(0.008)	(0.008)
Employment	−0.021^***^	−0.021^***^	−0.021^***^
	(0.004)	(0.004)	(0.004)
Cohort	−0.001	−0.001	−0.001
	(0.010)	(0.010)	(0.010)
Attitude gap	0.006^***^	0.006^***^	0.006^***^
	(0.001)	(0.001)	(0.001)
Smoking status	0.004	0.004	0.004
	(0.005)	(0.005)	(0.005)
Drinking status	−0.008	−0.008	−0.008
	(0.005)	(0.005)	(0.005)
Social interaction attitude	0.003	0.003	0.003
	(0.003)	(0.003)	(0.003)
Chronic status	0.059^***^	0.059^***^	0.059^***^
	(0.007)	(0.006)	(0.006)
Self-reported health	0.088^***^	0.088^***^	0.088^***^
	(0.002)	(0.002)	(0.002)
Inpatient	0.053^***^	0.053^***^	0.053^***^
	(0.005)	(0.005)	(0.005)
Log income	−0.002^***^	−0.002^***^	−0.002^***^
	(0.000)	(0.000)	(0.000)
Log savings	0.000	0.000	0.000
	(0.000)	(0.000)	(0.000)
Debt status	0.008	0.008	0.008
	(0.005)	(0.005)	(0.005)
Family size	−0.001	−0.000	−0.001
	(0.002)	(0.002)	(0.002)
Log GDP	−0.111^***^	−0.112^***^	−0.111^***^
	(0.027)	(0.024)	(0.024)
*F*-stat		1,513	2, 821
Underidentification test *P-*value		0.000	0.000
Year FE	Yes	Yes	Yes
Household FE	Yes	Yes	Yes
Provincial FE	Yes	Yes	Yes
Constant	0.889^***^		
	(0.270)		
Observations	88,144	88,144	88,144
*R* ^2^	0.851	0.084	0.084

^**^p < 0.05; ^***^p < 0.01.

Our control variables are also effective. We identify a non-linear relationship between age and mental health (87). Additionally, individuals with higher education levels, married people, males, and those employed tend to report better mental health. Physical health is positively associated with mental health (88). Conversely, individuals who perceive inequality as a serious issue tend to have poorer mental health. Additionally, higher income levels are positively correlated with better mental health outcomes (89).

In summary, Tables 2, 3 show that adverse consumption circumstances harm individuals' mental health. These findings contribute to the literature on relative deprivation related to consumption and mental health, specifically through the lens of hedonic consumption items.

### 4.2 Analysis of heterogeneity

We acknowledge that our instrumental strategy is not a panacea against all kinds of concerns. Therefore, we conduct heterogenous regressions to reassure results from different dimensions.

Chinese parents tend to spend substantial resources to their children particularly during their schooling years (ages 6–18). In addition to tuition, school-age children incur many unpredictable expenses, such as extracurricular tutoring, which reduces parents' hedonic spending. The intensity and frequency of expenditures on extracurricular tutoring are unpredictable, varying with different tutoring agencies and course demands. Additionally, the timing of extracurricular tutoring is uncertain, as parents typically enroll their children in tutoring only when their academic performance declines. Therefore, we assume that parents with children between the ages of 6 and 18 will suffer from higher levels of mental disorders due to expenditure uncertainty. The first two columns of Table 4 report the results, revealing that parents with children between the ages of 6 and 18 will suffer from higher levels of mental disorders, supporting Hypothesis 2a (the first column of Table 4 is the results that parents with children under the age of 6, while the second column of Table 4 is the result that parents with school-age children). Moreover, we identify the numbers of reduced hedonic consumption to examine Hypothesis 2b. The last two columns of Table 4 reveal that individuals experience greater distress when faced with sudden, one-time reduction rather than chronic reduction.

**Table 4:** The impact of predictable or unpredictable/one-time or chronic reduced hedonic consumption expenditure on mental health.

	**Predictable**	**Unpredictable**	**One-time**	**Chronic**
*Reduction*_*ft*_	0.011^**^	0.016^***^	0.016^***^	0.007
	(0.004)	(0.008)	(0.006)	(0.004)
Covariates	Yes	Yes	Yes	Yes
Fixed-effects	Yes	Yes	Yes	Yes
Observations	47,579	16,364	26,326	56,839
*R* ^2^	0.849	0.849	0.868	0.853

All models include fixed effects for individual, household, and year, plus all covariates. ^**^p < 0.05; ^***^p < 0.01.

Furthermore, we examine Hypothesis 2c from two perspectives. The first is the ratchet effect proposed by Duesenberry (40), and the second is that individuals facing economic hardships are more likely to involuntarily reduce consumption expenditure, especially hedonic consumption expenditure, thus, worsen mental health. The first column of Table 5 shows a pronounced impact on mental health when individuals experience a decline in their life situation compared to column 2. The findings of the last two columns show that individuals holding any debt are facing pronounced mental health than individuals holding saving in column 3 as indebtedness leads to consumption expenditure among hedonic items involuntarily. In sum, Table 5 supports Hypothesis 2c, illustrating that involuntary declines in hedonic consumption could result in greater mental disorders.

**Table 5:** The ratchet effect & The impact of reduced hedonic consumption expenditure on mental health among individuals with saving or indebtedness.

	**Model 1**	**Model 2**	**Saving**	**Indebtedness**
*Reduction*_*ft*_	0.015^**^	0.003	0.005	0.017^**^
	(0.006)	(0.006)	(0.004)	(0.007)
Covariates	Yes	Yes	Yes	Yes
Fixed-effects	Yes	Yes	Yes	Yes
Observations	22,032	21,310	55,784	30,168
*R* ^2^	0.877	0.875	0.863	0.874

All models include fixed effects for individual, household, and year, plus all covariates. ^**^p < 0.05.

Table 6 shifts focusing to the effects of consumption inequality on mental health, considering two dimensions: attitudes toward unfairness and experiences of unfair treatment. We divide the sample into two groups based on responses to the question: “How serious do you think the gap between the rich and the poor is in our country?” to measure attitudes toward unfairness. To assess experiences of unfair treatment, we use the following questions:

(1) Have you been treated unfairly due to differences between the rich and poor?

(2) Have you been treated unfairly because of your Hukou (household registration) status?

(3) Have you been treated unfairly due to your gender?

(4) Have you been treated unfairly by government officials?

**Table 6:** The impact of consumption inequality among hedonic items on mental health with different unfairness experiences.

	**Attitude: fairness**	**Attitude: unfairness**	**Experience unfairness: No**	**Experience unfairness: Yes**
*IneqC*_*ft*_	0.004	0.015^*^	0.003	0.021^**^
	(0.011)	(0.009)	(0.008)	(0.010)
Covariates	Yes	Yes	Yes	Yes
Fixed-effects	Yes	Yes	Yes	Yes
Observations	37,008	48,700	45,633	40,324
*R* ^2^	0.870	0.864	0.868	0.859

All models include fixed effects for individual, household, and year, plus all covariates. ^*^p < 0.1; ^**^p < 0.05.

We assign a value of 1 if the individual answers “yes” to any of these questions and 0 otherwise.

The results show that individuals who perceive or experience unfairness—whether real or perceived—report more severe mental health issues, supporting Hypothesis 2d.

### 4.3 Channels analysis

In this section, we conduct additional analysis to examine the channels through which reduced hedonic consumption expenditure and consumption inequality among hedonic items affect mental health.

Regarding the impact of reduced hedonic consumption expenditure, we argue that decreased trust plays a key mediating role. Panel A of Table 7 presents the results of the mediation analysis. Although Column (1) shows that reduced hedonic consumption expenditure lowers trust levels, this result is not statistically significant. However, the dataset provides detailed trust measures, including trust in strangers, parents, and neighbors (see the last three columns in Panel A). As expected, individuals tend to show less trust in strangers when experiencing a decline in hedonic spending. Therefore, Hypothesis 3a is supported.

**Table 7:** Channel analysis.

**Panel A**	**Trust levels**	**Trust in strangers**	**Trust in parents**	**Trust in neighbors**
*Reduction*_*ft*_	−0.005	−0.033^***^	−0.014	0.009
	(0.004)	(0.017)	(0.012)	(0.017)
Observations	79,015	78,865	78,883	79,119
*R* ^2^	0.290	0.312	0.306	0.337
Panel B	Social status	Income status		
*IneqC*_*ft*_	−0.019^*^	−0.024^**^		
	(0.011)	(0.012)		
Observations	116,792	111,929		
R^2^	0.303	0.317		

All models include fixed effects for individual, household, and year, plus all covariates. ^*^p < 0.1; ^**^p < 0.05; ^***^p < 0.01.

Similarly, for the impact of consumption inequality on mental health, we propose that diminished self-perceptions are a critical mediating factor. Panel B of Table 7 shows that consumption inequality significantly reduces self-reported social status and perceived income status, supporting Hypothesis 3b.

### 4.4 Moderating analysis

In this section, we perform additional analyses to examine the moderating effects on the relationship between relative deprivation related to consumption and mental health. The first two columns of Table 8 present the results for cultural variations as a moderator. The interaction term between religious beliefs and relative deprivation related to consumption shows a significantly negative sign on mental health. This indicates that religious beliefs can help mitigate mental health disorders. The last columns of Table 8 report the results for social capital as a moderator. These findings demonstrate that social capital reduces the negative impact of relative deprivation on mental health. Overall, the results support Hypotheses 4a and 4b.

**Table 8:** Moderating analysis.

	**Cultural variations**	**Cultural variations**	**Social capital**	**Social capital**
*Reduction*_*ft*_	0.011^***^		0.042^**^	
	(0.003)		(0.019)	
*IneqC*_*ft*_		0.016^**^		0.058^**^
		(0.006)		(0.025)
Religious beliefs	0.042^***^	0.053^***^		
	(0.008)	(0.010)		
$Reduction_{ft}^{*}$ Religious beliefs	−0.016^*^			
	(0.010)			
$IneqC_{ft}^{*}$ Religious beliefs		−0.032^**^		
		(0.013)		
Social capital			−0.010^***^	−0.008^***^
			(0.003)	(0.003)
$Reduction_{ft}^{*}$ Social capital			−0.003^*^	
			(0.002)	
$IneqC_{ft}^{*}$ Social capital				−0.004^*^
				(0.002)
Covariates	Yes	Yes	Yes	Yes
Fixed-effects	Yes	Yes	Yes	Yes
Observations	88,144	88,144	88,144	88,144
*R* ^2^	0.851	0.851	0.851	0.851

All models include fixed effects for individual, household, and year, plus all covariates. ^*^p < 0.1; ^**^p < 0.05; ^***^p < 0.01.

### 4.5 Robustness checks

In the robustness analysis, we test our results using different independent variables. To explore the relationship between reduced consumption expenditure and mental health, we replace reduced hedonic consumption with experiential consumption expenditure. For consumption inequality, we substitute the Kakwani index with the Gini coefficient and Theil index.

Experiential purchases are defined as “those made with the primary intention of acquiring a life experience,” such as trips, eating out, and movies, which tend to bring more happiness than material purchases. In terms of consumption inequality, existing studies commonly measured using macro-level indices like the Gini coefficient or Theil index.

Table 9 reports the findings. In Panel A, consumption inequality among hedonic items, as measured by both the Gini coefficient and Theil index, significantly worsens mental health. Panel B shows that reduced experiential consumption expenditure also significantly harms mental health, despite the first column of Panel B is insignificant. In sum, Table 9 demonstrates that different independent variables do not change the basic conclusion of this paper.

**Table 9:** Robustness checks.

**Panel A**	**IV**	**Lewbel's IV**	**IV**	**Lewbel's IV**
*IneqC*_*ft*_	0.201^***^	0.038^***^	0.143^***^	0.014^*^
	(0.055)	(0.019)	(0.025)	(0.008)
*F*-stat	179	1,007	163	1,213
Observations	87,272	87,272	87,272	87,272
*R* ^2^	0.082	0.084	0.078	0.084
Panel B	IV	Lewbel's IV		
*Reduction*_*ft*_	0.012	0.006^**^		
	(0.007)	(0.003)		
*F*-stat	424	7,972		
Observations	77,925	77,925		
*R* ^2^	0.088	0.088		

All models include fixed effects for individual, household, and year, plus all covariates. ^*^p < 0.1; ^**^p < 0.05; ^***^p < 0.01.

## 5 Conclusions and discussions

The relative deprivation related to consumption in recent years has ignited intense debate as COVID-19 pandemic caused incalculable economic losses worldwide. The global economy is still facing great uncertainty in the post-pandemic era, with rising unemployment and falling wages and incomes as problems for governments around the world, which undoubtedly weigh on consumption figures. Concurrently, the pandemic contributed to the escalation of economic inequality around the globe in unprecedented ways. Consumption, as an important indicator to measure the well-being of residents, needs special attention.

This paper utilizes representative Chinese data to examine the impact of relative deprivation related to consumption on mental health from two perspectives: vertical comparisons of reduced hedonic consumption expenditure and horizontal comparisons of consumption inequality among hedonic items. Our findings indicate that decreased spending on hedonic goods worsens mental health, primarily by reducing trust levels. Additionally, consumption inequality among hedonic items negatively impacts mental health by diminishing self-perceptions, leading to psychological distress. Furthermore, moderating effects show that religious beliefs, as well as social capital could diminish the negative effect of relative deprivation related to consumption on mental health.

Our findings support the existing literature on the impact of economic hardship on mental health issues such as indebtedness, poverty, and income loss (89–91), despite our research builds on this literature by focusing on reduced hedonic consumption. Furthermore, our data reveal that individuals who experience sudden, involuntary reductions in hedonic consumption face a higher risk of mental disorders. Regarding inequality, previous research has linked income inequality, wealth inequality, and rural-urban inequality to worsened mental health (34, 92, 93), and our study expands on this by examining consumption inequality among hedonic items. Notably, we find that experiences of unfair treatment amplify the negative mental health effects of consumption inequality. Within the specific cultural and technological context of China, this study uncovers several critical insights. First, the Confucian tradition of “face culture”, emphasizing social recognition and approval, inevitably influences consumption behavior. For example, China's hedonic consumption expenditure ranks among the highest globally due to face culture. However, when economic hardships lead to reduced consumption, individuals may feel they have lost face, resulting in psychological burdens. Second, with the continuous development of China's internet industry and the increasing prevalence of smartphones and tablets, people are now more exposed to sharing and witnessing hedonic moments on social media than ever before. While social media usage is often considered a way to reduce social isolation and loneliness (94), entertainment-oriented platforms can trigger comparisons through the sharing of hedonic content, exacerbating mental health issues.

Additionally, we explored potential channels. As for reduced consumption expenditure, Chinese people have unconditional trust in their own family (the core “in-group”), relatively to the people who is not in one's family called the “out-group” (95), explaining the significance of decreased trust in strangers sever as a crucial mediator. Consumption inequality as group relative deprivation is personal inferior status perceived from social comparison. When an individual feels this unfavorable social comparison, he may have a more negative self-evaluation or low self-perceptions, highlighting the importance of diminished self-perceptions as a critical mediator.

Furthermore, this paper examines the moderating roles of religious beliefs and social capital. Although religious beliefs in China exhibit certain unique characteristics (96), the study finds that 22% of respondents identify as religious, and 36% engage in some form of religious activities, such as ancestor worship, exorcism, or divination (97). As religious activities rapidly expand, their impact on Chinese culture, society, and the economy is becoming increasingly evident, with more individuals seeking psychological solace through religion (98). Meanwhile, social capital, an essential concept in the social sciences, has gained considerable attention in public health research (99). From a “micro” perspective, existing studies indicate that higher levels of social capital can effectively mitigate the adverse effects of mental health problems (100).

Based on the above findings, the following implications were made for alleviating mental disorder: (1) Stabilize the Economic Outlook. Raising income levels and encouraging consumption, particularly on hedonic goods, can improve mental health. Government efforts to stabilize economic expectations and increase residents' incomes through effective distribution mechanisms and other measures are essential. For example, Long et al. suggest that optimizing income distribution boosts household consumption, especially by raising the income levels of low-income groups (101). (2) Addressing Socioeconomic Vulnerabilities. The COVID-19 pandemic has highlighted societal vulnerabilities and exacerbated pre-existing socioeconomic inequalities, disproportionately affecting health outcomes. The government should focus on supporting vulnerable populations, including unmarried individuals, those with lower education levels, the unemployed, residents in underdeveloped areas, and individuals facing declining incomes and over-indebtedness. Additionally, expenses related to child-rearing and healthcare can reduce hedonic consumption. Hence, building a more effective safety net is crucial. (3) Promoting Social Equity. Narrowing the wealth gap and promoting social equity are vital for improving residents' mental health. The government should implement policies aimed at reducing income inequality and fostering a more equitable society. For example, the development of the digital economy helps reduce income inequality (102), enhance social equality (103), and boost household consumption, particularly in the areas of developmental and hedonic spending (104). (4) Strengthen Social Engagement and Community Networks. Advocating for traditional cultural activities, such as festival celebrations, folk arts, or meditation classes, can enhance a sense of community belonging and provide a sense of purpose. Additionally, developing multifunctional community centers to foster inclusive social activities and strengthen interpersonal connections can also contribute to better mental health.

This study has several strengths. First, our sampling frame represents 95% of the Chinese population, providing national representativeness. Second, our large sample size (88,144 in baseline regression) ensures high statistical power and considerable external validity. Third, the use of instrumental variable analysis addresses concerns of endogeneity.

Despite its strengths, our study also suffers from limitations. First, the findings are primarily based on China's unique economic and cultural context. Issues of relative deprivation in consumption in China are driven by distinct factors such as high savings rates, Confucian traditions, and regional development imbalances. Caution is advised when generalizing these results to countries with different economic structures and cultural contexts. Future research could conduct replication studies in varied settings to enhance external validity. Second, our independent and dependent variables are derived from different levels, despite prior studies employing the same identification. We suggest that future data collection efforts incorporate consumption behaviors and mental health status at the individual level. Third, while the instrumental variable approach mitigates endogeneity issues, residual bias or confounding variables may persist. Future research could employ experimental designs to further enhance validity. Fourth, we use the Kessler 6 rating Scale and the Center for Epidemiologic Studies Depression Scale to measure mental health in our study. However, mental health is a broad concept. Future research should replicate our study using specialized datasets, such as The Psychology and Behavior Investigation of Chinese Residents dataset (105).

In conclusion, this study advances our understanding of how relative deprivation related to consumption affects mental health and offers valuable insights for policymakers and practitioners aiming to address these challenges.

## Data Availability

The original contributions presented in the study are included in the article/supplementary material, further inquiries can be directed to the corresponding author.

## Footnotes

^1^The list of the CESD 20 items can be found at Center for Epidemiologic Studies Depression Scale (CES-D) (brown.edu).
